# Radiation in Veterinary Practice: Paradigm Shift Toward Precision and Curative Approaches

**DOI:** 10.3390/life16040626

**Published:** 2026-04-08

**Authors:** Sorin Marian Mârza, Camelia Munteanu, Radu Lăcătuş, Ionel Papuc, Florin Dumitru Bora, Robert Cristian Purdoiu

**Affiliations:** 1 Clinical Sciences Department, Faculty of Veterinary Medicine, University of Agricultural Sciences and Veterinary Medicine Cluj-Napoca, 3-5 Manastur Street, 400372 Cluj-Napoca, Romania; radu.lacatus@usamvcluj.ro (R.L.); ionel.papuc@usamvcluj.ro (I.P.); robert.purdoiu@usamvcluj.ro (R.C.P.); 2 Biology Section, Faculty of Agriculture, University of Agricultural Sciences and Veterinary Medicine Cluj-Napoca, 3-5 Manastur Street, 400372 Cluj-Napoca, Romania; camelia.munteanu@usamvcluj.ro; 3 Laboratory of Chromatography, Advanced Horticultural Research Institute of Transylvania, Faculty of Horticulture and Business for Rural Development, University of Agricultural Sciences and Veterinary Medicine Cluj-Napoca, 3-5 Manastur Street, 400372 Cluj-Napoca, Romania; boraflorindumitru@gmail.com

**Keywords:** radiation oncology, external beam radiotherapy, brachytherapy, targeted radionuclide therapy, stereotactic radiosurgery, stereotactic body radiotherapy, radiosyviorthesis, low-dose radiotherapy, comparative oncology

## Abstract

Ionizing radiation therapy has undergone a clear paradigm shift in veterinary oncology and inflammatory disease management, moving from mainly palliative use toward structured, curative treatment programs. This review synthesizes current evidence on key modalities used in veterinary practice, including external beam radiotherapy, brachytherapy, systemic targeted radionuclide therapy, stereotactic radiosurgery, stereotactic body radiotherapy, radiosynoviorthesis, and low-dose radiotherapy. Each modality is discussed in relation to its physical and biological basis, major isotopes or beam types, routes of delivery, target species such as dogs, cats, and horses, clinical indications, and global availability. Comparative analysis highlights differences in clinical acceptance, evidence strength, access, and cost. External beam radiotherapy and stereotactic techniques support curative tumor management, whereas radiosynoviorthesis and low-dose radiotherapy are effective for inflammatory and degenerative disorders. Despite ongoing progress, challenges remain in access, dosimetry standardization, and prospective evidence. Companion animals are also emphasized as valuable translational models, guiding future innovation and collaboration internationally.

## 1. Introduction

Ionizing radiation therapy has become an indispensable tool in the management of cancer in companion animals, evolving from an occasional palliative measure into a spectrum of techniques that can offer durable local control and, in selected cases, curative intent. Historically, radiotherapy in veterinary practice began as simple superficial x-ray treatments for skin lesions and pain relief. Over the last two decades, advances in imaging, planning software and delivery systems have driven a rapid expansion of precise external-beam and implantable techniques for dogs and cats [1].

The change in clinical mindset from predominantly palliative use to more frequent definitive, organ-sparing treatment plans reflects three parallel developments. First, modern linear accelerators and planning systems permit conformal dose distributions that spare adjacent normal tissues; second, evidence from multi-institutional veterinary series shows meaningful survival and quality-of-life gains for select tumor types when definitive courses are used; and third, comparative oncology has emphasized the translational value of treating naturally occurring cancers in companion animals as a bridge to human oncology [2].

In early veterinary practice, radiotherapy was most often applied to reduce pain, control bleeding, or shrink bulky masses where surgery was not feasible. Such uses prioritized short, hypo-fractionated regimens to limit visits and side effects [1]. Over time, however, fractionation concepts and better imaging allowed clinicians to escalate doses to tumors while maintaining acceptable toxicity, enabling definitive-intent protocols for brain, nasal, oral and some soft tissue tumors in dogs and cats [2]. Stereotactic techniques and image guidance have accelerated the trend toward organ-preserving, high-precision therapy. Localized lesions, stereotactic body radiotherapy (SBRT) or stereotactic radiosurgery (SRS) now represent viable alternatives to surgery [3].

At the cellular level, ionizing radiation kills or disables malignant cells primarily by inducing DNA lesions, with double-strand breaks (DSBs) being the most lethal form when unrepaired or misrepaired. The DNA damage response governs cell fate after irradiation and determines radiosensitivity; understanding these pathways has facilitated translational approaches and radiosensitization strategies in veterinary comparative oncology [4]. Clinically, the goal of radiotherapy is to maximize the therapeutic ratio—that is, to deliver a tumoricidal dose while limiting dose to surrounding normal tissues through choices in fractionation, beam modulation and image guidance [5].

Different modalities exploit physical and biological principles to optimize this ratio. Conformal external-beam approaches such as three-dimensional conformal radiotherapy (3DCRT), intensity-modulated radiotherapy (IMRT) and volumetric modulated arc therapy (VMAT) shape dose distributions tightly around targets, thereby allowing dose escalation or normal-tissue sparing [2]. Stereotactic methods deliver very high biologically effective doses in few fractions, exploiting steep dose gradients to protect adjacent organs. In veterinary series, SBRT has shown promising local control for selected pulmonary and soft-tissue tumors [3]. Brachytherapy, where radioisotopes are placed within or next to tumors, offers an alternative route to deliver focal high doses while reducing integral dose to the patient [6].

Since 2000, research in veterinary radiation oncology has grown rapidly, closely reflecting developments in human medicine and building a stronger evidence base for IMRT, SBRT, brachytherapy, and image-guided techniques. Yet differences in reporting practices, dosimetry, fractionation, and outcome measures continue to make comparisons across studies challenging [5]. A recent systematic appraisal of stereotactic techniques emphasized variability in technical reporting and underlined the need for standardized outcome metrics in veterinary series [7]. Given these gaps, an up-to-date synthesis that compares modalities by intent (palliative versus definitive), biologic rationale, practical delivery and clinical outcomes is timely. The present review aims to summarize the major modalities used in companion animals, evaluate contemporary clinical evidence, and highlight methodological limitations that should guide future trials and reporting.

## 2. Materials and Methods

### 2.1. Study Design

The goal of this study, which was carried out as a narrative synthesis with systematic components, was to compile the most recent research on radiation modalities for companion animals. The PRISMA (Preferred Reporting Items for Systematic Reviews and Meta-Analyses) standards were followed in structuring the process to improve transparency and reproducibility.

### 2.2. Method of Search

Three electronic databases—PubMed/MEDLINE, Web of Science, and Embase—were thoroughly searched for relevant material. The search encompassed research that was published between January 2000 and December 2025 in order to capture recent advancements in radiation oncology for animals.

Boolean operators (AND, OR) were used in search techniques to mix free-text keywords with regulated vocabulary (MeSH terms). “Veterinary radiotherapy,” “external beam radiotherapy,” “3D-CRT,” “IMRT,” “VMAT,” “stereotactic radiosurgery,” “SRS,” “stereotactic body radiotherapy,” “SBRT,” “brachytherapy,” “targeted radionuclide therapy,” “radiosynoviorthesis,” “dogs,” “cats,” and “companion animals” were among the primary search terms.

The following database filters were used:

Language: English

Species: dogs and cats (if relevant)

Type of publication: peer-reviewed journal articles

Period: 2000–2026

To find more relevant studies, the reference lists of pertinent publications and reviews were also manually examined.

### 2.3. Selection of Studies

**Figure 1:**
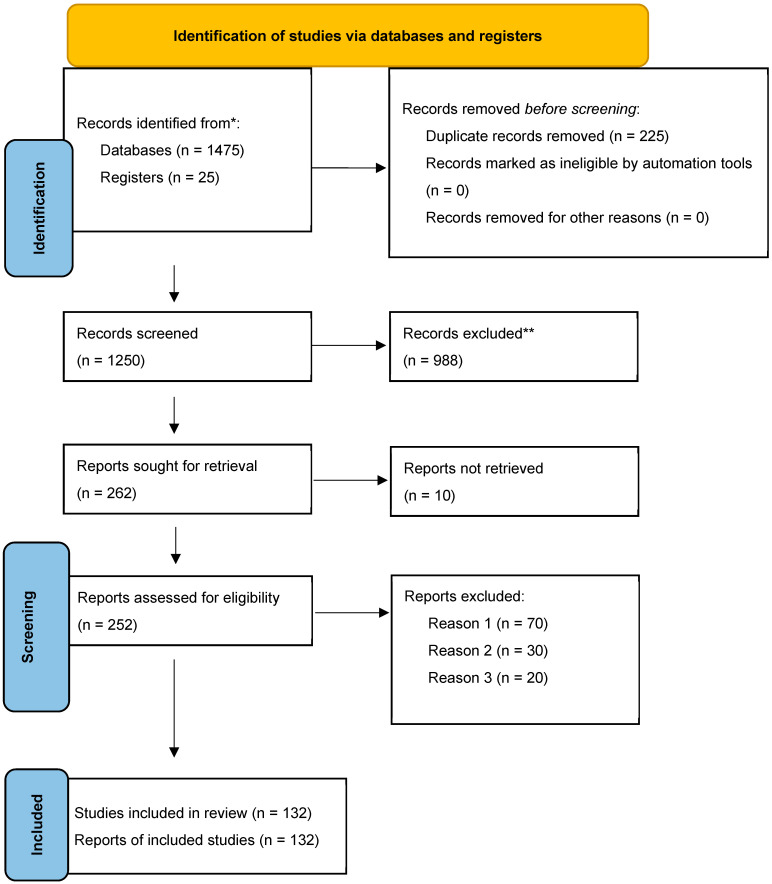
* Consider, if feasible, reporting the number of records identified from each database or register searched (rather than the total number across all databases/registers). ** If automation tools were used, indicate how many records were excluded by a human and how many were excluded by automation tools. Source: https://www.bmj.com/content/372/bmj.n160 [8] (accessed on 21 March 2026). This work is licensed under CC BY 4.0. To view a copy of this license, visit https://creativecommons.org/licenses/by/4.0/ (accessed on 21 March 2026).

Figure 1 summarizes the study selection procedure, which adhered to PRISMA guidelines. Duplicate records that were found through database searches were eliminated and exported. A full-text evaluation of possibly suitable studies was conducted after titles and abstracts were screened for relevance.

Criteria for inclusion:

Peer-reviewed publications in journals with indexes

Research on companion animals (cats and dogs)

Clinical or translational research detailing radiotherapy’s effects, toxicity, dosimetry, or technological features

Meta-analyses, systematic reviews, and clinical or observational studies

Criteria for exclusion:

Case studies with insufficient dosimetric or technical information

Conference abstracts that are not available in complete text

Publications without peer review, or “grey literature”

Research unrelated to radiation

Articles written in languages other than English

The most thorough or recent publication was used when overlapping study populations were found.

### 2.4. Data Synthesis and Extraction

Data were manually retrieved and classified according to the type of radiation. Study design, sample size, species, tumor type, treatment modality, fractionation schemes, dosimetry, clinical outcomes, and reported toxicities were among the variables that were extracted.

Quantitative meta-analysis was not possible because of the variability in study designs, tumor types, and treatment methods. As a result, a qualitative synthesis was carried out, classifying the findings according to treatment intent (palliative versus definitive) and modality.

### 2.5. Evaluation of Evidence Quality

A modified hierarchy of evidence was used to evaluate the quality of the included research. Research was categorized as follows:

High level: prospective multi-institutional research, meta-analyses, and systematic reviews.

Moderate level: well-planned retrospective cohorts and prospective single-institution studies.

The study design, sample size, follow-up period, reporting consistency, and dosimetric detail were used to qualitatively assess the risk of bias [9].

### 2.6. Flow Diagram for PRISMA

A PRISMA flow diagram (Figure 1) shows the number of records found, screened, excluded, and included at each stage of the literature selection process.

### 2.7. Restrictions

The heterogeneity of the research, the scarcity of randomized controlled trials, and the inconsistent reporting criteria are the primary limitations of this analysis. Direct comparisons between radiation methods are limited by these factors.

### 2.8. Preparing Figures

Figures were created in BioRender. Munteanu, C. (2026) https://BioRender.com/01kpeif and https://BioRender.com/mgg6ot9 (accessed on 16 February 2026).

## 3. External Beam Radiotherapy (EBRT)

External beam radiotherapy (EBRT) denotes the delivery of ionizing radiation from a machine external to the patient, most commonly using high-energy photon or electron beams produced by a medical linear accelerator (linac) [10]. Linacs accelerate electrons, which may be used directly for the treatment of superficial targets or made to strike a high-Z target to produce bremsstrahlung photons for deeper-seated lesions; clinical selection between electrons and photons depends on tumor depth, required dose distribution and organ-at-risk proximity [10].

Three-dimensional conformal radiotherapy (3D-CRT) uses CT-based planning and shaped beams to cover the planning target volume (PTV) while lowering the dose to surrounding tissues relative to older two-dimensional approaches [11]. In veterinary practice, 3D-CRT remains widely used for both definitive and palliative intents, since it reliably improves dose conformity with comparatively modest increases in planning complexity [2]. For tumors such as nasal carcinomas and certain oral masses, 3D-CRT permits fractionated protocols that balance local control with acceptable late-effect risk [12]. Intensity-modulated radiotherapy (IMRT) modulates beam fluency across multiple beamlets to sculpt dose distribution tightly around complex targets, which often achieves superior sparing of nearby critical structures [5]. Veterinary multi-institutional series report that IMRT reduces ocular and neurologic toxicities in intranasal and cranial tumors while maintaining tumor coverage, supporting its increasing adoption at referral centers [2].

Volumetric modulated arc therapy (VMAT) delivers IMRT-like modulation dynamically while the gantry rotates, shortening treatment time and often improving organ-at-risk sparing compared with step-and-shoot IMRT [13]. Dosimetry comparisons in veterinary targets have shown that VMAT reduces the dose to lacrimal structures and the cornea in sinonasal plans, which can translate into lower acute and late ocular toxicity when clinical workflows permit its safe application [14]. Shorter beam-on times make VMAT attractive for animal patients that are anesthetized, lowering anesthesia duration and associated risks [13]. EBRT is a mainstay for many canine tumors, with clear indications including nasal carcinomas, intracranial neoplasms, mast cell tumors in incompletely excised sites, and certain soft tissue sarcomas where local control dictates outcome [15]. Definitive fractionation regimens (e.g., 18–20 fractions of 2.5–3 Gy) are commonly used for curative intent in intracranial or sinonasal disease, whereas coarse hypo-fractionated protocols (e.g., 4–6 fractions of 6–9 Gy or single-fraction stereotactic approaches) are selected for palliation or stereotactic treatments [16]. Studies of SBRT for primary pulmonary and selected soft tissue tumors report good local control and acceptable toxicity, offering an alternative to surgery in anatomically challenging cases [3]. In cats, EBRT is frequently used for cutaneous and oral squamous cell carcinoma, certain nasal tumors and selected intracranial lesions; hypo-fractionated regimens are common because of patient and owner considerations [17]. Postoperative finely fractionated protocols following cytoreductive surgery have demonstrated improved local control in specific feline head-and-neck indications [16].

In equine medicine, EBRT has been applied mainly to periocular and cutaneous tumors such as sarcoid and squamous cell carcinoma, as well as selected head lesions; however, the need for specialized stabling, equipment and anesthesia logistics renders EBRT less common than in small animal practice [18]. Common oncologic indications treated with EBRT across species include mast cell tumors (adjuvant or definitive), nasal and oral carcinomas, primary brain tumors, and soft tissue sarcomas [15]. Definitive protocols are designed to maximize local control and typically use daily fractions over several weeks, while palliative programs use larger fractions and fewer visits to rapidly relieve signs with acceptable short-term morbidity [19,20,21]. While 3D-CRT is the most broadly available EBRT technique in veterinary clinics, IMRT and VMAT are increasingly established at tertiary referral centers and university hospitals where physics resources permit their safe use [14,21].

Stereotactic techniques (SRS/SBRT) have transitioned from experimental to mainstream in many referral settings, supported by growing clinical series documenting favorable control rates for select indications [5]. Advanced EBRT services for animals are concentrated in countries with established veterinary oncology centers; notable regions include North America (USA, Canada), Western Europe (UK, Germany, Switzerland, and Netherlands), Japan and selected institutions across Australia and parts of East Asia [22,23]. In lower-resource settings, EBRT may be absent or limited to older kilovoltage systems, which constrains access to definitive-intent, highly conformal therapies [10,24,25].

## 4. Localized Internal Radiotherapy (Brachytherapy)

Brachytherapy is the direct placement of radioactive sources within or next to a tumor to deliver a high, localized radiation dose while sparing surrounding normal tissues [22]. This approach contrasts with external beam delivery by minimizing the path length through healthy tissue and by producing very steep dose gradients around the implanted sources [19]. Clinically, brachytherapy can be delivered as low-dose-rate (LDR) permanent implants, as temporary high-dose-rate (HDR) after loading, or as intermediate schemes; each format leverages radiobiological differences in dose rate and fractionation to balance tumor kill against late normal-tissue effects [26]. In veterinary patients, the principal advantage is the ability to give a large tumoricidal dose directly to the resection bed or residual disease with a short overall treatment time and limited whole-body exposure [27].

Common radionuclides used for interstitial and intracavitary brachytherapy include Iridium-192 for HDR after-loading and Iodine-125 for permanent or temporary low-dose implants; Cesium-131 has more recently been explored for select intracranial and soft-tissue indications [28,29]. Iridium-192 sources are typically delivered via remote after-loading units using flexible catheters placed at surgery or through percutaneous tunnels, permitting precise dwell-time programming and rapid dose delivery [30]. Iodine-125 seeds are often embedded in stranded form or implanted individually under image guidance, providing continuous low-dose irradiation that is advantageous for head-and-neck sites [26]. Interstitial techniques place sources directly within the tumor volume, while intracavitary approaches position sources inside a natural lumen adjacent to the lesion, such as the nasal or oral cavity, to exploit proximity for dose conformity [31].

Device development has included flexible after-loading catheters, three-dimensional treatment planning with CT or MRI fusion, and shielded remote after-loaders that improve staff safety while enabling HDR schedules that compress treatment into days rather than weeks [32]. These technical advances allow for higher precision, reproducible source geometry, and improved documentation of delivered dose, which are essential when adapting human brachytherapy principles to veterinary practice [19]. Brachytherapy in veterinary medicine is used primarily in dogs and cats, with applications including oral and oropharyngeal squamous cell carcinoma, transmissible venereal tumor, nasal tumors, and feline injection-site sarcoma as an adjuvant to surgery [27]. For feline injection-site sarcoma, interstitial Iridium-192 implants placed at the time of tumor excision have been reported to deliver effective local doses with acceptable complication rates, particularly where wide surgical margins are limited by anatomical constraints [27]. In dogs, HDR interstitial programs for intranasal tumors have demonstrated promising local control and symptom relief in early series, suggesting that brachytherapy may serve as an organ-preserving alternative to radical surgery in selected cases [30].

Iodine-125 permanent seed implantation has been explored experimentally and clinically for accessible oral tumors and salvage settings, where continuous low-dose delivery and reduced need for repeated anesthesia offer practical benefits [26]. For tumors located in superficial or surgically challenging regions, brachytherapy may be used as a focal boost to external beam radiotherapy or, in limited cases, as a standalone definitive modality [33]. Its use in equine patients remains uncommon, although isolated reports describe the treatment of superficial and periocular tumors where shallow dose penetration helps protect deeper ocular and neural structures [34]. Brachytherapy remains a specialized technique within veterinary oncology, yet it is an established option at selected tertiary referral centers and academic hospitals with radiation oncology infrastructure [19]. The current evidence base consists largely of pilot prospective investigations and translational research using companion animals, rather than large randomized trials, which limits formal guideline development and broad standardization [27]. Ongoing research focuses on improved applicator design, enhanced image guidance including intraoperative CT and cone-beam CT, and evaluation of alternative isotopes such as Cesium-131 for tight-margin or intracranial applications, where shorter half-life characteristics may be advantageous [28]. Technological innovations such as three-dimensional printed templates for precise needle placement and real-time dosimetry verification are being explored to reduce operator variability and expand clinical access [32]. Future directions include multicenter registries to harmonize outcome reporting, comparative studies against modern external beam techniques for matched indications, and investigation of combined-modality approaches integrating brachytherapy with immunotherapy or radiosensitizing agents [19]. At present, brachytherapy occupies a focused but valuable role in cases where anatomy, prior surgery, or the need for rapid and localized dose delivery makes it the preferred option.

## 5. Systemic Radiotherapy (Targeted Radionuclide Therapy)

Targeted radionuclide therapy (TRT) delivers ionizing radiation to disease sites after the systemic administration of a radiopharmaceutical that selectively accumulates in target tissue [35]. The therapeutic construct combines a radioactive isotope with a targeting moiety, a small molecule, peptide or antibody, so that the radiation dose is concentrated where the biological target concentrates the vector [36]. Mechanisms of uptake include bone affinity for phosphonate or calcium-seeking agents, receptor-mediated internalization for peptide or antibody ligands, and transporter activity for iodide analogues. The emitted particles (beta, alpha or Auger) determine the path length and radiobiological effect [37].

Bone-seeking beta emitters such as Samarium-153-EDTMP and Strontium-89 have a long history in human bone-pain palliation and have been evaluated in dogs with osteosarcoma and other painful bone lesions [38]. Recent veterinary phase I and pilot trials of Sm-153 formulations in companion dogs reported measurable reductions in pain and improved mobility with generally manageable marrow toxicity when monitored [39]. Strontium-89 remains an option for durable palliation in selected cases, although its longer half-life and marrow dose profile require cautious selection and monitoring [35].

Radioiodine (I-131) is the archetypal systemic radiopharmaceutical in veterinary practice and is considered the standard, often curative, therapy for feline hyperthyroidism because hyperactive functional thyroid tissue preferentially takes up iodide [40]. Large series demonstrate high rates of biochemical remission and acceptable complication rates after a single therapeutic administration, establishing I-131 as a benchmark for safe systemic therapy in companion animals [41].

Emerging agents in human nuclear oncology, notably Lutetium-177 and Yttrium-90 labeled peptides and antibodies, have motivated translational evaluation in veterinary patients and models, expanding targets to receptor-positive tumors and enabling locoregional radio-embolization approaches [42,43]. Preclinical and early clinical labeling studies indicate these payloads can be stably conjugated to targeting vectors and maintain biological activity in canine models, supporting further clinical evaluation [44].

TRT is most commonly administered by intravenous injection, and systemic biodistribution depends on the pharmacokinetics of the targeting moiety and the radionuclide’s physical properties such as half-life and emission type [36]. Veterinary dosimetry presents special challenges because standard human biokinetic models do not scale directly to dogs and cats, inter-animal uptake variability is large, and few validated species-specific models exist [19]. Reliable absorbed-dose estimation therefore commonly requires serial nuclear imaging (planar, SPECT/CT or PET/CT when available) combined with blood and urine sampling to derive subject-specific kinetics and to limit marrow and organ exposure [36,45].

Regulatory and practical constraints create additional barriers; clinics must maintain licensed radiopharmacy support, shielding and waste protocols, and patient-release criteria that meet national regulations, which limits wider adoption in general practice [46]. Clinically, myelosuppression is the principal dose-limiting toxicity for beta emitters; therefore, pre-treatment hematology, dose adjustments and longitudinal monitoring are standard requirements in veterinary protocols [37].

Dogs and cats are the principal species treated clinically or enrolled in TRT trials, and they provide important translational models because many of their spontaneous tumors recapitulate human histology and tumor microenvironments [47]. Dogs with osteosarcoma have been treated with bone-seeking agents in palliative trials and with locoregional Y-90 approaches in proof-of-concept series, generating data on feasibility, dosimetry and safety that inform both veterinary practice and comparative oncology [48]. Cats remain the primary beneficiaries of I-131 therapy for hyperthyroidism, with well-described treatment pathways and outcomes that serve as a model for systemic radionuclide therapy delivery and post-release criteria [40].

Today, TRT in veterinary oncology is predominantly palliative or investigational; radioiodine for feline hyperthyroidism is the clearest curative example, while bone-seeking agents and newer radiopharmaceuticals are mainly used for symptom control or within clinical trials [35]. Translational research is broadening available isotopes and vectors and exploring combination approaches that pair TRT with immunotherapy or external-beam radiation to potentiate immune-mediated effects and local control [42].

Key barriers to broader clinical adoption include regulatory complexity, constrained veterinary radiopharmacy supply chains for small-batch doses, the need for multidisciplinary teams including nuclear medicine-trained veterinarians and physicists, and the costs of imaging and individualized dosimetry [49]. Nevertheless, recent pilot and phase I studies of Sm-153 formulations and canine Y-90 radio-embolization demonstrate that TRT can be delivered safely and can provide symptom relief and functional benefit when performed in well-resourced centers [48].

Priority next steps are the development and validation of veterinary-specific dosimetry models, creation of multicenter outcome registries to harmonize efficacy and toxicity reporting, and development of commercially available veterinary formulations to simplify dosing and regulatory compliance [19]. Continued collaboration between veterinary oncology, nuclear medicine and medical physics will be essential to expand TRT options and to translate novel agents safely into clinical practice for animals with cancer [44].

## 6. Stereotactic Radiosurgery (SRS) and Stereotactic Body Radiotherapy (SBRT)

Stereotactic radiosurgery (SRS) and stereotactic body radiotherapy (SBRT) refer to radiation techniques that deliver very high biological doses to well-defined targets in one or a few treatment sessions with submillimeter to millimeter precision, thereby maximizing tumor dose while limiting exposure to adjacent normal tissues [50]. Historically developed for intracranial lesions, the stereotactic paradigm has been extended to extracranial sites as SBRT, which uses similar geometric precision but is adapted to the respiratory and soft-tissue motion of the body [51]. The central concept is that steep dose gradients and small target margins allow ablative or near-ablative biologically effective doses to be delivered safely, which in turn can produce rapid and durable local control for many tumor histologies [52]. SRS and SBRT are therefore defined by the combination of immobilization, imaging, planning, and delivery accuracy rather than by a single machine or dosimetry prescription.

Successful stereotactic treatments require high-quality image guidance, commonly cone-beam CT (CBCT) or stereoscopic X-ray systems, to verify patient position immediately before or during each fraction [7]. Rigid immobilization for cranial SRS and body frames or reproducible thermoplastic and vacuum devices for SBRT reduce setup error and support the tight margins necessary for stereotactic dose distributions [53]. Advanced planning systems that support small-field dosimetry, heterogeneity correction, inverse optimization and robust collision avoidance are central to producing clinically acceptable plans for complex shapes and organs at risk near the target [54]. Motion management is often required for thoracic and abdominal lesions and may combine breath-hold, gating, or target tracking strategies to maintain dosimetry precision across the respiratory cycle [50]. High dose rates and flattening-filter-free (FFF) delivery modes can reduce beam-on time and therefore the duration of anesthesia in veterinary patients, which is important because most animals require sedation or general anesthesia for SRS/SBRT delivery [55]. Quality assurance (QA) programs that include end-to-end testing, daily imaging QA and small-field dosimetry validation are non-negotiable prerequisites for safe stereotactic practice [7].

Most veterinary SRS and SBRT programs use high-energy photon beams produced by medical linear accelerators, commonly in the 6 MV range but sometimes extending to 10 MV depending on machine configuration and treatment depth [56]. Flattening-filter-free 6 MV beams are attractive because they offer high dose rates and favorable penumbra characteristics for small fields used in stereotactic work [55]. The choice of photon energy must balance penumbra, buildup, out-of-field dose and neutron production (relevant for very high energies), and most veterinary centers therefore favor 6 MV or 6 MV FFF delivery for intracranial and many extracranial targets [10].

Advanced delivery platforms used in veterinary practice include gantry-based linacs with multileaf collimators for VMAT/arc SBRT and robotic systems that permit non-coplanar beam trajectories for cranial SRS. Dosimetry accuracy for very small fields used in SRS is technically demanding and relies on small-field detector calibration, rigorous commissioning and regular QA checks [7].

Intracranial SRS and hypofractionated stereotactic protocols are widely used for primary brain tumors in dogs, including meningiomas and gliomas, delivering single-fraction or few-fraction high doses that yield meaningful tumor shrinkage, symptom relief and prolonged median survival times versus historical controls [54,57]. SRS has proven effective for pituitary-dependent acromegaly and for selected small intracranial lesions in cats, with reports of endocrine and neurologic control following treatment [58]. For sinonasal tumors in dogs, SBRT regimens such as 3 × 10 Gy have been reported in large cohorts with good early local control and acceptable toxicity profiles, making SRT a commonly chosen definitive or palliative option for otherwise challenging anatomy [12]. Thoracic SBRT has been applied to primary pulmonary tumors and metastatic lung nodules in dogs, producing encouraging local control rates and low acute morbidity in multiple single-institution series [59]. SBRT has also been used successfully for vertebral and paraspinal lesions to achieve pain relief and local disease control while limiting spinal cord dose with steep gradients and careful planning [59]. For cutaneous and subcutaneous tumors such as injection-site sarcomas in cats, hypofractionated stereotactic approaches have served as a useful salvage or palliative tool, particularly where resection was limited by location or comorbidity [60,61]. Larger cohorts now provide more robust estimates of survival, patterns of failure and toxicity than early case reports, yet heterogeneity in fractionation and reporting remains a barrier to pooled meta-analysis [7].

Fractionation schemes vary widely and are chosen according to tumor histology, location, size and proximity to critical structures, with single-fraction SRS typically reserved for small intracranial targets and 1–5 fraction SBRT schemes used for extracranial sites [62]. Common veterinary prescriptions include 1 × 18–24 Gy for small intracranial targets or 3 × 8–12 Gy for many body sites, but clinicians frequently adapt dose to organ-at-risk constraints and patient tolerance [63,64]. Biological effective dose calculations and normal tissue constraints drawn from human experience are often used as a starting point, but veterinary-specific data and dose–response relationships are emerging and should inform planning whenever available [54].

Across indications, stereotactic approaches produce high rates of objective response and symptom palliation while maintaining acceptable acute toxicity, but late effects, particularly neurologic decline after cranial SRT, have been reported and require careful long-term follow-up [65,66]. Reported median survival times for dogs with intracranial meningiomas treated with SRS/SRT commonly exceed one year, and subsets of patients experience multi-year control, which supports the role of stereotactic therapy as a curative-intent option in selected cases [54,67]. Sinonasal SBRT series show good early control but variable late morbidity that correlates with dose to ocular and nasal structures, emphasizing the need for meticulous planning and accurate dose reporting [12]. Comparative studies indicate that SBRT can provide outcomes similar to more protracted definitive protocols for some tumors while reducing anesthesia time and cost, though randomized data are lacking [68,69].

SRS and SBRT capability for animals is concentrated in tertiary referral hospitals and university centers with access to modern linacs, image-guided systems and physics support, a distribution pattern that mirrors human radiotherapy infrastructure globally [53]. Regions with notable activity include North America, Western Europe, Japan and Australia, where multiple centers report caseloads, QA protocols and outcomes in peer-reviewed journals [7]. Growth in stereotactic use has been rapid over the past decade, driven by improved software, motion management, availability of FFF beams and growing clinical experience reported in numerous studies. Survey data reveal substantial heterogeneity in techniques, imaging protocols and dose reporting among practitioners, which has prompted calls for standardized reporting checklists and multi-center registries to harmonize datasets and improve comparability [53,70]. International collaborations and consensus efforts are underway to refine veterinary stereotactic dose constraints, reporting items and QA tolerances, with the goal of making outcome data more interoperable and clinically actionable [7].

Active research in veterinary stereotactic radiation includes investigations of combined modality therapy with immunotherapy, radiobiologic studies of dose-per-fraction effects in spontaneous tumors, and development of adaptive and MRI-guided workflows to further shrink planning margins and improve normal tissue sparing [71,72,73]. Additional work explores lattice and spatially fractionated stereotactic concepts, dose painting for hypoxic tumor subvolumes, and the application of radionics and perfusion imaging to predict early response and toxicity [74,75]. The literature also highlights the need for veterinary-specific dose–volume constraints, prospective registries and randomized comparisons where feasible to define when stereotactic approaches should replace or complement conventional fractionation [76]. Key practical constraints include the need for daily or intrafraction imaging, resources for robust anesthesia and motion management, and the financial and logistical investments required for commissioning and maintaining stereotactic systems [7]. In many regions, access is limited by equipment availability and workforce capacity, and referral decisions must balance potential clinical benefit against travel and cost burdens borne by owners [53]. Lastly, caution is advised when extrapolating human normal-tissue limits directly to veterinary patients because species-specific tolerance and differences in life expectancy and comorbidity may alter the risk-benefit calculus [54].

## 7. Radiosynoviorthesis (Radiation Synovectomy)

Radiosynoviorthesis (RSO), also termed radiosynovectomy or radiation synovectomy, is a minimally invasive nuclear medicine procedure in which a beta-emitting radiocolloid is delivered directly into the joint space to irradiate and ablate inflamed synovial lining cells and macrophages, thereby reducing synovial hypertrophy, effusion and pain [77]. The rationale for the procedure rests on selective local radiation delivery: particulate radiocolloids are phagocytosed by synovial macrophages and retained in the subsynovial tissue, where emitted beta and conversion electrons produce localized cytotoxic effects with a steep dose fall-off that spares most extra-articular structures [78,79].

RSO was developed as an alternative to surgical synovectomy and to steroid injections for persistent, treatment-refractory synovitis, and it is valued for its outpatient nature, relatively low complication rate and potential to delay or avoid more invasive interventions [80]. The clinical effect is achieved by inducing focal synoviocyte necrosis and by reducing the inflammatory cellular burden, which can interrupt the cycle of synovitis and secondary joint damage that drives symptoms in both inflammatory and degenerative joint diseases [78]. In veterinary medicine, the same mechanistic rationale applies, but species-specific joint anatomy, particle clearance and patient handling require adapted dosimetry, release criteria and procedural safeguards [59]. Interest in RSO for animals has increased because chronic synovitis is a major contributor to pain and dysfunction in osteoarthritis (OA) and immune-mediated joint disease, and because many canine and feline patients are poor candidates for long-term systemic therapies [81].

Recent translational studies have therefore sought to quantify feasibility, biodistribution, clinical response and owner-reported outcomes after intra-articular radiocolloid administration in dogs, which serve as both target patients and comparative models for human therapy [82]. The three classical beta-emitting radiocolloids used in human RSO are Yttrium-90 (^{90}Y) for large joints such as the knee, Rhenium-186 (^{186}Re) for medium joints such as the shoulder and wrist, and Erbium-169 (^{169}Er) for small joints of the hand and foot; each isotope is selected for its particle energy, tissue penetration and half-life [77].

Yttrium-90 emits high-energy beta particles with a tissue range appropriate for the large synovial volumes of the knee, and its clinical track record in human rheumatoid and osteoarthritic knees underpins its widespread use [83]. Rhenium-186 has an intermediate beta energy and is commonly used for medium-sized joints where deeper penetration than erbium is needed but a shorter range than yttrium is preferred to limit extra-articular exposure [84]. Erbium-169 emits low-energy beta particles and is therefore suited to small joints, where shallow penetration reduces risk to surrounding tissues and where the synovial surface area is limited [85].

In veterinary practice, a newer option has emerged: a homogeneous tin-117m (^{117m}Sn) colloid that emits conversion and Auger electrons with very short path lengths, offering highly focal synovial irradiation and minimal external exposure, and which is being developed and evaluated specifically for canine OA [82]. Preclinical biodistribution and dosimetry work has demonstrated retention of ^{117m}Sn particles in canine joints with minimal leakage, and subsequent pilot and controlled clinical studies have reported clinical benefits in elbow OA and other target joints. The choice of agent in a given clinical case therefore depends on the joint size, desired penetration depth, radioisotope half-life, availability and regulatory approval in the jurisdiction where the procedure will be performed [77,86].

RSO is performed under strict aseptic and radiation-safety conditions and begins with accurate joint aspiration to remove effusion and to diminish the joint volume prior to radiocolloid injection [77]. Imaging guidance, such as ultrasound or fluoroscopy, is frequently used to ensure intra-articular needle placement, particularly in anatomically difficult joints or in small animals where blind palpation is unreliable [87]. After aspiration and verification of needle position, the radiocolloid is injected slowly, the needle is flushed when protocol dictates, and the joint is gently mobilized to distribute the colloid uniformly across the synovial surface [77]. Corticosteroid co-injection is sometimes used in human practice to mitigate acute post-procedural synovitis, but veterinary protocols vary and the decision balances potential short-term benefit against interference with radiocolloid phagocytosis [78]. Techniques for preventing extra-articular leakage include careful needle positioning, post-injection imaging (planar scintigraphy or SPECT), and in some protocols temporary immobilization of the joint; these measures inform post-procedure release criteria and public-safety guidance [83].

Because dogs and cats are normally anesthetized or heavily sedated for joint injection, attention to anesthesia risk, sterile technique and post-anesthesia monitoring are integral parts of veterinary RSO workflows [59]. Post-procedure surveillance includes periodic clinical assessment, owner pain-score instruments (e.g., canine Brief Pain Inventory), and, when available, imaging follow-up to document retention and to exclude extra-articular migration [81]. The principal veterinary species treated with RSO are dogs, and the most common indication in current practice and trials is elbow osteoarthritis and associated synovitis, although stifle and shoulder joints have also been targeted [82]. Canine osteoarthritis often involves a significant synovial-inflammatory component; RSO seeks to reduce this inflammation and enhance limb function and quality of life, especially when systemic therapy is not feasible or insufficient [88]. Clinical pilot studies and randomized cohorts in dogs have reported meaningful reductions in owner-assessed pain, improvements in weight bearing and extended intervals of functional benefit after a single intra-articular dose of ^{117m}Sn colloid [89].

Repeat injections at roughly annual intervals have been shown to be feasible and to produce sustained benefit in many patients, with safety profiles that compare favorably to chronic systemic analgesic use when proper precautions are observed [81]. There is current but limited experience with RSO in cats and in large animals; feline indications would logically include severe monoarticular OA or synovitis where other options fail, but evidence in cats remains sparse compared with dogs [29]. Beyond OA, RSO has theoretical application in immune-mediated monoarthritis and in hemophilic arthroplasty in animals, mirroring human practice, but such uses in veterinary patients are currently exploratory and dependent on case selection and regulatory clearance [68]. In human medicine, RSO is well established in many European countries, particularly Germany, where national guidelines and approved products for ^{90}Y, ^{186}Re and ^{169}Er exist and where the procedure remains in routine use for refractory mono- and oligoarthritis [77]. Similarly, clinical experience and regulatory pathways in Germany and Italy underlie relatively broad adoption in Europe, while practice in North America has been historically limited by regulatory and reimbursement differences and by reduced clinician familiarity with RSO [80].

Veterinary uptake mirrors the human geographical pattern: early and sustained use in Europe is paralleled by a growing but more cautious adoption in North America, where ^{117m}Sn products and veterinary-specific safety guidance have only recently enabled broader clinical trials and marketing authorizations [90]. Regulatory controls, nuclear medicine infrastructure, and the need for licensed handling and disposal pathways currently limit widespread availability to tertiary referral hospitals and specialty practices in higher-resource regions [87]. Nevertheless, prospective randomized and controlled veterinary studies over the last decade have strengthened the evidence base for canine RSO, and multicenter registries are now being proposed to harmonize outcome measures, adverse-event reporting and dosimetry standards [82]. Economic and client-acceptance considerations also drive adoption; owners often prefer a single, long-acting local therapy to chronic oral medications, which has increased demand for safe, effective RSO agents and for transparent release and radiation-safety guidance in clinical practice [82].

Systematic reviews and consensus guidelines in human medicine conclude that RSO provides clinically meaningful benefit for resistant synovitis with an acceptable safety profile, and they recommend isotope selection and procedural standards to optimize outcomes [77]. In veterinary practice, the strongest and most reproducible evidence presently supports the use of ^{117m}Sn colloid in canine elbow OA, with multiple prospective and randomized studies reporting improved objective and owner-reported outcomes and low procedure-related morbidity [82]. Safety data emphasize minimal systemic exposure and low external dose rates with appropriate product formulations and technique, but they also highlight the need for species-specific release criteria and age-dependent dose-rate considerations to protect owners and staff [90]. Important research priorities include validating species-specific dosimetry models that translate particle energy and retention into absorbed synovial dose estimates, performing longer-term follow-up for potential late effects, and comparing RSO head-to-head with alternative intra-articular therapies in randomized trials [91,92]. Advances such as 3D-printed injection templates, image-guided delivery, and improved particle designs are helping make treatments safer and extend the range of joints that can be treated [32]. Finally, coordinated multicenter registries and shared reporting standards modeled on EANM guidance in human care will be essential to produce sufficiently powered datasets that can define indications, dose–response relationships and comparative effectiveness for veterinary RSO [77].

## 8. Low-Dose Radiotherapy (LDRT) for Inflammatory Conditions

Low-dose radiotherapy (LDRT) refers to radiotherapeutic regimens that deliver very small doses per fraction, typically on the order of 0.5–1.0 Gy per fraction, or cumulative low total doses intended to elicit anti-inflammatory and analgesic effects rather than direct cytotoxic tumor control [93]. The clinical rationale for LDRT is rooted in consistent clinical observations across decades, showing symptom relief in patients with painful degenerative and inflammatory musculoskeletal disorders after low-dose courses—an effect now examined at the molecular and cellular level [94].

**Figure 2:**
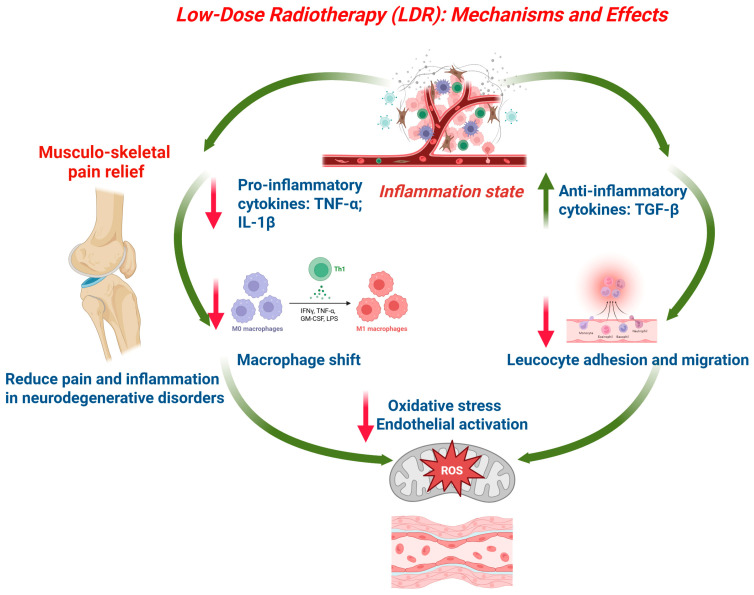
Mechanisms and biological effects of low-dose radiotherapy (LDR). Low-dose radiotherapy helps regulate the inflammatory environment by reducing pro-inflammatory cytokines (e.g., TNF-α, IL-1β) and increasing anti-inflammatory mediators (e.g., TGF-β). This promotes a shift in macrophages toward an anti-inflammatory state. As a result, leukocyte adhesion and migration are reduced, endothelial activation is limited, and oxidative stress (ROS production) is diminished. Together, these effects help calm the inflammatory response and are associated with clinical benefits such as pain relief in musculoskeletal conditions and reduced inflammation in neurodegenerative disorders.

At the cellular scale, low doses of ionizing radiation modulate inflammatory cell behavior by altering cytokine expression, reducing leukocyte adhesion and migration, shifting macrophage phenotypes toward anti-inflammatory states, and influencing endothelial function; together, these changes can reduce tissue edema and pain [95]. Experimental models indicate that LDRT decreases the production of pro-inflammatory mediators such as TNF-α and IL-1β, while upregulating regulatory cytokines like TGF-β, which may promote a local environment conducive to the resolution of inflammation rather than persistent tissue damage [96] (Figure 2). Additional mechanistic hypotheses propose dose-dependent redox modulation, transient alterations in nitric oxide synthase activity, and effects on cell adhesion molecules that collectively reduce the recruitment and activation of inflammatory cells at the irradiated site [97]. There is emerging evidence that LDRT may also induce systemic immunomodulatory signals following local irradiation, with reports of distant anti-inflammatory effects in animal models, suggesting that local LDRT can influence whole-organism inflammatory set points [94]. Mechanistic clarity remains incomplete, however, because the dose–response relationships are non-linear and time-dependent, and because differing endpoints (cell signaling, gene expression, tissue histology, clinical pain) respond on different timescales and with variable sensitivity to dose and dose rate [93].

Consequently, mechanistic research is active and seeks to translate observed cellular and molecular effects into rationally designed clinical LDRT protocols that maximize benefit and minimize long-term risk [98]. Historically, LDRT for benign inflammatory conditions has been delivered with orthovoltage X-ray units or with low-energy electron beams because these modalities deposit dose superficially and can be tailored to the depth of inflamed tissue typical in joints and periarticular structures [99]. Common fractionation schemes in human practice vary by indication and center but often use single fractions of 0.5–1.0 Gy delivered daily or on alternate days for cumulative doses of about 3–6 Gy, or slightly higher regimens depending on the disease and historical practice patterns [100]. Orthovoltage (kilovoltage) devices remain widely used in Europe for superficial LDRT because of their dosimetry suitability for skin and shallow joint targets, while megavoltage linac-based electrons can be chosen when slightly greater penetration or beam shaping is required [49]. Treatment planning for LDRT is typically simpler than for high-dose oncologic plans, but proper field definition, shielding of adjacent tissues, and attention to reproducible setup are still essential to ensure the intended low dose reaches the inflamed volume and that cumulative dose constraints are respected [101]. Protocols in veterinary practice have mirrored human approaches with adaptations for species differences, and they commonly use orthovoltage units or megavoltage electrons to treat joints such as the canine elbow and stifle with single or few low-dose fractions [102]. Because many veterinary patients require sedation or anesthesia to maintain immobilization during treatment, practitioners sometimes adopt fractionation schemes that limit the number of sessions while still delivering a biologically appropriate low total dose [103]. Dosimetry reporting in both human and veterinary series increasingly emphasizes absorbed dose to the target, beam quality, field size and number of fractions to enable reproducible protocols and support pooled analyses across centers [104].

The principal veterinary application of LDRT to date is the management of degenerative joint disease, especially osteoarthritis of the elbow and stifle in dogs, where synovitis and periarticular inflammation are core contributors to pain and dysfunction [102]. Pilot studies and clinical series report improvements in objective measures such as weight-bearing and owner-reported pain scores after single- or few-fraction low-dose courses, with many animals experiencing sustained benefits for several weeks to months following treatment [105]. A 2016 veterinary study reported that a single low dose produced short-term improvements in weight bearing in dogs with elbow osteoarthritis, demonstrating feasibility and suggesting clinical effect sizes that warrant larger controlled trials [103]. An Italian center reported a series of dogs treated with LDRT for OA and recorded clinical benefit with minimal acute toxicity, supporting the translational link between human LDRT experience and veterinary practice [102]. Beyond OA, low-dose approaches have been trialed for inflammatory skin conditions, keloids, and hyperactive proliferative benign lesions in animals, and orthovoltage therapy is particularly suited to cutaneous targets because of its superficial dose deposition [99]. There are isolated reports of LDRT being used for benign soft-tissue masses and perianal adenomas in dogs where surgical options are limited or recurrence is problematic, and these case series document symptomatic or local-control benefits in selected patients [17]. Comparative data are still limited, but emerging evidence from multicenter veterinary cohorts and translational trials using spontaneous canine disease suggests that LDRT can be a useful adjunct when medical management fails or when owners seek alternatives to chronic systemic therapy [105]. Safety signals in veterinary series are reassuring: acute skin irritation is uncommon with well-planned orthovoltage fields, and late radiation-induced effects appear rare at the low total doses typically used for benign indications, though long-term surveillance remains important [102]. LDRT continues to be more commonly used and accepted in parts of Europe than in North America, a pattern driven by historical practice, guideline support from European societies, and greater clinical familiarity in those regions [101]. European recommendations and consensus statements provide structured indications, suggested fractionation schedules and risk-mitigation strategies that underpin continuing clinical uptake for conditions such as degenerative joint disease and certain soft-tissue disorders [104].

Nevertheless, controversy persists around LDRT because randomized controlled trial evidence is limited relative to the historical observational data, and because the theoretical carcinogenesis risk, though small at therapeutic low doses, remains a point of debate that influences guideline conservatism and patient selection [100]. Systematic reviews of human LDRT studies indicate that numerous trials demonstrate symptomatic benefit, though study designs differ, highlighting the need for contemporary randomized trials with standardized endpoints and modern radiotherapy techniques to address remaining uncertainties [106,107]. Veterinary debate mirrors the human field: proponents highlight clinical improvements, low acute toxicity and the single-session convenience for animal patients, while critics emphasize small sample sizes, variable outcome measures and the need for long-term safety data before widespread adoption [105].

Regulatory and practical considerations also influence use: orthovoltage units are less common in some regions, and linac-based low-dose treatments require careful beam selection and dosimetry to reproduce historical orthovoltage experience, which complicates multicenter standardization [49]. Guideline bodies therefore recommend careful case selection, informed consent that includes discussion of uncertain long-term risks, and registry enrollment or trial participation when feasible to build the evidence base [101]. Recent translational work and renewed clinical interest, prompted in part by improved understanding of immune modulation by low radiation doses, have stimulated new trials and multicenter registries in both human and veterinary medicine that aim to produce higher-quality evidence [94]. Finally, while LDRT is not a panacea, it represents a pragmatic, low-toxicity option for selected animal patients with chronic, treatment-refractory inflammatory conditions, and its appropriate role will be clarified as pooled data, randomized trials and standardized reporting accumulate [102].

## 9. Comparative Analysis and Synthesis

Ionizing radiation is used as therapy in veterinary medicine, and there are many modalities that vary in mechanism or mode of use, target species, and clinical maturation.

Localized internal radiotherapy, or brachytherapy, involves high-dose irradiation of the target tissue, which is localized with minimal exposure to other structures.

RSO is an inflamed synovium that is treated with the beta-emitting radionuclides of Yttrium-90, Erbium-169 and Tin-117m using intra-articular injection. Low-dose radiotherapy (LDRT), which incorporates orthovoltage or low-energy evanescent beams, is an anti-inflammatory and pain reliever that uses a sub-therapeutic (less than 1 Gy per fraction) dose of ionizing radiation.

**Figure 3:**
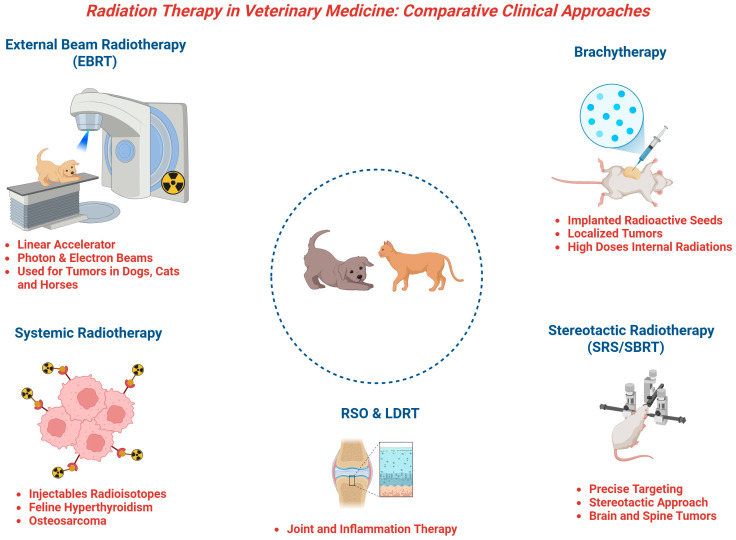
Radiation therapy in veterinary medicine: comparative clinical approaches. This figure presents the main radiation therapy options used in companion animals. External beam radiotherapy (EBRT) uses linear accelerators to deliver photon or electron beams and is commonly applied in the treatment of tumors in dogs, cats, and horses. Brachytherapy involves placing radioactive sources directly within or close to the tumor, allowing for highly targeted radiation delivery. Systemic radiotherapy relies on injectable radiopharmaceuticals and is used in conditions such as feline hyperthyroidism and certain bone tumors. Stereotactic radiotherapy (SRS/SBRT) enables very precise, high-dose treatment, particularly for brain and spinal tumors. In addition, low-dose radiation therapy (LDRT) is used for non-cancer conditions, including joint and inflammatory diseases. Together, these approaches reflect the diversity of radiation-based treatments available in modern veterinary oncology.

The strength of evidence depends on EBRT and SRS/SBRT, which have been assessed in numerous clinical series reporting tumor response, survival, and safety in dogs and cats [108,109] (Figure 3).

The accessibility, cost, specialization, and evidence strength synthesis help to emphasize the choice of therapeutic modality according to the clinical contexts and available resources. Whereas the use of EBRT and SRS/SBRT prevails in oncology centers, RSO and LDRT provide viable, cheaper, and less invasive alternatives for chronic inflammatory and degenerative diseases and improve the quality of life for patients with minimal systemic toxicity (Table 1). Head-to-head comparisons, standardized outcome reporting, and translational studies should be the priorities of future research to maximize the selection of modalities and introduce radiotherapy into veterinary practice in a more comprehensive way [95,102].

## 10. Discussion

### 10.1. Major Advances and Trends: Integration of Advanced Imaging, Hypofractionation, and Growing Access

Over the last two decades, veterinary radiotherapy has shifted from largely palliative, superficial treatments toward precise, organ-sparing and sometimes definitive approaches, driven by three intersecting technological trends. First, the routine incorporation of cross-sectional imaging CT and MRI fusion has markedly improved the anatomical definition of tumors and adjacent organs at risk, enabling tighter margins and more confident dose escalation in anatomically complex sites [110]. Second, image guidance at the time of treatment using cone-beam CT or stereoscopic radiography has reduced setup error, which permits the adoption of stereotactic concepts and hypofractionated regimens that shorten overall treatment time and reduce anesthetic exposure for animal patients [111]. Third, modern beam-modulation techniques such as intensity-modulated radiotherapy and volumetric-modulated arc therapy, together with small-field stereotactic delivery systems, have allowed clinicians to sculpt dose distributions around irregular targets and spare critical structures that previously limited definitive treatment [30]. Implementation of flattening-filter-free beams and high dose-rate delivery has further reduced beam-on time, which is a tangible safety and logistical benefit in a species that requires anesthesia for most treatments [112]. Collectively, these advances have legitimized the wider use of stereotactic radiosurgery and stereotactic body radiotherapy in tertiary veterinary centers, with many institutions reporting encouraging local control and symptom relief in intracranial, sinonasal and pulmonary tumors following 1–5 fraction regimens [113]. Importantly, the translation of these methods has required rigorous commissioning, small-field dosimetry validation and daily quality assurance to ensure that the steep dose gradients characteristic of stereotactic plans are delivered accurately and safely [112].

Further details on delivery innovations is warranted because they directly shape clinical outcomes. Flattening-filter-free beams and higher dose rates reduce beam-on time and thus anesthesia duration, which is a practical advantage in veterinary patients and lowers anesthetic risk during ablative treatments [112]. Small-field dosimetry advances, including the use of micro-chambers, radiochromic film and solid-state detectors, have improved accuracy for stereotactic fields and allowed greater confidence in delivering steep dose gradients near critical structures [30]. At the same time, the adoption of volumetric imaging for adaptive workflows permits plan modification in response to target shrinkage or weight changes, which is particularly relevant for head and neck or thoracic tumors, where anatomy can change across weeks of treatment [110]. Evidence supporting hypofractionation in selected veterinary tumor types has grown, with single-fraction radiosurgery and short SBRT courses achieving promising median survival times and symptom control in several institutional series [113]. To illustrate clinical impact, intracranial meningiomas and presumptive gliomas in dogs treated with stereotactic or hypofractionated techniques commonly achieve median survival times that exceed historical expectations for untreated animals, providing meaningful extensions of quality of life and neurologic function in many cases [114]. Sinonasal tumor series also show that modern megavoltage and IMRT-based protocols improve local control compared with historical orthovoltage treatments, and that careful dose constraints of ocular structures can reduce late morbidity [115]. In thoracic disease, SBRT applied to primary lung tumors or metastatic nodules in dogs has produced high local control with limited acute toxicity in multiple institutional reports, making it a viable alternative when surgery is not feasible [113]. These favorable outcomes have encouraged referral centers to invest in stereotactic capability, but such investments demand sustained caseloads and institutional commitment to QA and interdisciplinary care [112].

### 10.2. Translational Potential: How Veterinary Patients Serve as Spontaneous Disease Models for Human Oncology (Comparative Oncology)

Companion animals offer distinctive translational value because their cancers arise spontaneously in immunocompetent, outbred hosts and therefore recapitulate key biological complexities, such as heterogeneity, microenvironmental interactions and natural metastatic behavior that are difficult to model in rodents [19]. This similarity enables veterinary trials to answer mechanistic and clinical questions that are directly relevant to human oncology, including dose–response relationships, normal tissue tolerance and immune modulation after radiotherapy [116]. For example, canine trials combining focal radiotherapy with immunomodulatory agents have provided early evidence that radiation can potentiate systemic immune responses and that sequencing and fractionation influence the likelihood of abscopal effects, thereby informing human immunoradiotherapy designs [117]. Comparative studies have also used molecular imaging and serial dosimetry in dogs to validate imaging biomarkers of radioresistance and to develop individualized dosing strategies that are applicable to human trials [118]. Moreover, interventional radionuclide techniques such as yttrium-90 radio-embolization and lutetium-labeled agents have been piloted in canine patients to establish feasibility, dosimetry and acute toxicity profiles before clinical translation, accelerating the bench-to-bedside cycle [119]. Companion animals furthermore enable endpoints that matter to owners, such as functional measures, pain scores and quality-of-life instruments that are often difficult to capture robustly in early human trials, thereby enriching translational interpretation and patient-centered decision making [118]. When combined with contemporary imaging and molecular assays, such data can refine selection criteria for human trials and prioritize agents most likely to produce meaningful clinical benefits in real-world settings [110].

### 10.3. Limitations and Challenges: High Cost, Limited Global Access, Need for Specialized Training, and Gaps in Evidence-Based Protocols

Despite clear technical progress and translational promise, several practical and scientific barriers restrict broader adoption and rigorous evaluation of advanced radiotherapy in veterinary medicine. Economically, the purchase and upkeep of linacs, imaging systems and brachytherapy after-loaders, together with shielding and radiopharmacy requirements for systemic therapies, create high fixed costs that limit the number of centers able to offer these services [120]. Evidence generation remains difficult because the literature is dominated by clinical and single-institution series, making pooled meta-analyses challenging and impeding the definition of species-specific dose–volume constraints and long-term toxicity benchmarks [118]. Regulatory heterogeneity for therapeutic radionuclides, disposal pathways and patient release criteria further hampers multicenter trials and the routine clinical use of systemic radiopharmaceuticals in animals [119]. Finally, owner preferences, cost constraints and the need for repeated anesthesia shape clinical decisions in ways that differ from human practice, limiting the external validity of some comparative findings and requiring trial designs that reflect real-world owner-centered outcomes [120].

From a systems viewpoint, global analyses quantifying human radiotherapy shortfalls are instructive for veterinary strategists because they reveal how regional investment, training hubs and shared resources can amplify reach and sustainability while preserving safety and regulatory compliance [120]. Translating these lessons into veterinary service design will require close collaboration with human radiation oncology and national regulatory authorities.

### 10.4. Future Directions: Prospects for Novel Radiopharmaceuticals, Immunoradiotherapy, Artificial Intelligence in Planning, and Global Collaboration to Standardize Care

Several areas offer particularly high potential for advancing both veterinary care and translational science. Novel radiopharmaceuticals and radioimmunoconjugates, including lutetium and yttrium constructs linked to tumor-selective ligands, are moving rapidly in human oncology and have practical feasibility data in canine models that can guide dose selection and safety monitoring [119]. Combining focal radiation with immunotherapies or tumor vaccines is another promising approach because radiation can enhance antigen presentation and modify the tumor microenvironment to favor systemic immune responses, a synergy observed in both preclinical work and early clinical reports [116,117]. Artificial intelligence and knowledge-based planning tools can address critical bottlenecks by automating segmentation, predicting achievable dose distributions and standardizing plan quality across centers, which is especially important in settings with limited physics support [121,122,123]. Adoption of shared registries, common reporting checklists for stereotactic and brachytherapy series, and harmonized toxicity grading will increase data interoperability and enable pooled analyses that produce robust, evidence-based recommendations for species-specific practice [118,124]. Finally, global initiatives to expand access through tele-physics, regional training hubs and international guidelines can reduce inequities in care and support ethical, high-quality multicenter clinical research that advances both animal welfare and human oncology [125].

At the research front, clarifying the optimal sequencing and fractionation of combined immunoradiotherapy remains a high priority because emerging data indicate that timing and dose per fraction influence whether radiation promotes systemic antitumor immunity or immunosuppression [126]. Equally, the role of low-dose radiotherapy for inflammatory conditions warrants rigorous comparative trials in veterinary cohorts because human randomized data are mixed and species differences in biology and lifespan may alter risk–benefit calculations [100,127].

Practical recommendations for the next research phase are straightforward and actionable. First, the development of prospective, multicenter registries with harmonized data fields for patient signalmen, tumor histology, imaging biomarkers, dose–volume metrics and standardized toxicity grading would enable pooled analyses that are currently impossible from single-center series [118]. Second, investment in training curricula that combine didactic modules, supervised clinical fellowships and remote mentorship will help expand the workforce while maintaining safety standards, and these curricula should be developed in collaboration with human radiation oncology and medical physics stakeholders to leverage existing expertise [125]. Third, research into cost-effective models of care, such as regional centers of excellence, shared remote planning services, and tele-QA networks, can reduce per-care costs and improve access while conserving high-complexity resources for cases that most benefit from them [120,128]. Fourth, rigorous prospective trials that compare hypofractionated stereotactic regimens with conventionally fractionated protocols for common tumor types are needed to define trade-offs in local control, late toxicity and quality of life in species-specific contexts [115]. Fifth, AI tools must be validated prospectively in veterinary cohorts and integrated with human expert oversight; automated contours and plans should be audited against expert consensus and clinical outcomes before routine deployment [121,124]. Finally, ethical and regulatory frameworks governing veterinary radiopharmaceutical use should be harmonized to enable multicenter phase I and II studies that can inform both veterinary standards of care and human translational pathways [119].

Contemporary veterinary radiotherapy is at a pivotal point in which technological capability, comparative relevance and clinical need converge. With careful stewardship, the next decade can deliver more precise, accessible and evidence-based radiotherapy for animals and, in turn, provide high-quality comparative data that accelerate beneficial innovations for human patients [48].

## 11. Conclusions

Ionizing radiation has become a central tool in the management of cancer in companion animals, offering interventions that range from single-fraction palliative courses to highly conformal, multi-fraction regimens designed for durable local control [129]. Despite these technological gains, access to modern radiotherapy remains uneven across regions and practice types, since high-performance linear accelerators, stereotactic delivery systems and experienced medical physicists are concentrated in referral and academic centers [129]. Economic and workforce constraints therefore shape which animals receive advanced care and limit the external validity of many single-center outcome series, creating a need for more inclusive clinical infrastructure and support programs [130].

From a scientific standpoint, substantial knowledge gaps persist, most notably incomplete species-specific normal tissue tolerance data and limited veterinary dose–response curves for common tumor histologies, which complicate the confident adoption of aggressive hpofractionation protocols [117]. Similarly, systemic radiopharmaceuticals and emerging theranostic agents lack validated, species-tailored pharmacokinetic and dosimetry platforms, which constrains personalized activity prescriptions and increases uncertainty about marrow and organ exposure in dogs and cats. Nevertheless, new modalities provide genuine opportunities to improve outcomes.

Theranostic strategies and targeted radioligand therapies form a parallel translational avenue; experience with lutetium-labeled agents in human oncology provides a regulatory and methodological template, and companion animal studies can yield biodistribution and toxicity data that inform both veterinary care and human trials. Multicenter trials that harmonize contouring, dose metrics and toxicity grading will support the derivation of species-appropriate dose–effect models and help define normal tissue constraints for common clinical targets. Registries that collect long-term outcomes and late effects will be especially valuable for detecting rare adverse events and for refining practice guidelines on an evidence basis [129].

Regulatory adaptation is also required; workable licensing and release criteria for veterinary radiopharmaceuticals and clear environmental stewardship rules will facilitate evidence-based clinical trials while safeguarding public and occupational health. Ethical oversight should be embedded in both clinical practice and comparative research, with informed consent processes that disclose likely benefits, alternatives and uncertainties, and trial designs that include validated quality-of-life instruments and humane stopping rules [117].

Cross-disciplinary consortia that unite veterinary clinicians, medical physicists, nuclear medicine experts and radiobiologists are particularly well-placed to design pragmatic trials that address clinically relevant questions efficiently and ethically [53]. Work on automated planning and AI-assisted contouring may accelerate workflow and reduce inter-observer variability, but such tools must be validated on veterinary imaging sets before deployment [109].

Pursuing these coordinated scientific, educational, and regulatory priorities will allow radiotherapy to develop as a humane, effective, and translationally valuable tool in the care of companion animals with cancer while simultaneously advancing comparative oncology knowledge that benefits human patients [131]. The coming decade therefore offers a realistic prospect that improved access, standardized evidence and ethical practice will increase treatment options and outcomes for animals and deepen the reciprocal benefits of veterinary and human radiation research [53]. Stakeholder engagement must remain an ongoing priority [132].

## Figures and Tables

**Table 1:** Summary of ionizing radiation therapies in veterinary medicine.

Therapy Type	Key Agents	Main Indications	Clinical Status
EBRT	Photon/electron beams	Solid tumors (MCT, nasal, brain, sarcoma)	Established
Brachytherapy	Ir-192, I-125	Localized tumors (oral, TVT, nasal)	Specialized
Systemic Radiotherapy	Sm-153, Sr-89, I-131	Osteosarcoma, hyperthyroidism	Palliative/Emerging
SRS/SBRT	High-energy photons	Intracranial, spinal, soft tissue tumors	Advanced/Growing
RSO	Y-90, Er-169, Sn-117m	Osteoarthritis, polyarthritis	Regional (EU-focused)
LDRT	Low-energy X-rays	Inflammatory/degenerative conditions	Controversial

## Data Availability

No new data were created or analyzed in this study. Data sharing is not applicable to this article.

## Abbreviations

The following abbreviations are used in this manuscript:

3D-CRT	Three-Dimensional Conformal Radiotherapy
AI	Artificial Intelligence
CBCT	Cone-Beam Computed Tomography
CT	Computed Tomography
DSBs	Double-Strand Breaks
EBRT	External Beam Radiotherapy
FFF	Flattening-Filter-Free
FLASH	(Refers to ultra-high dose rate radiotherapy)
HDR	High-Dose-Rate
IGRT	Image-Guided Radiotherapy
IL-1β	Interleukin-1 Beta
IMRT	Intensity-Modulated Radiotherapy
LDR	Low-Dose-Rate
LDRT	Low-Dose Radiotherapy
linac	Linear Accelerator
MLCs	Multileaf Collimators
MRI	Magnetic Resonance Imaging
OA	Osteoarthritis
PET/CT	Positron Emission Tomography/Computed Tomography
PRRT	Peptide Receptor Radionuclide Therapy
PTV	Planning Target Volume
QA	Quality Assurance
RSO	Radiosynoviorthesis (also called Radiation Synovectomy)
SBRT	Stereotactic Body Radiotherapy
SPECT	Single-Photon Emission Computed Tomography
SRS	Stereotactic Radiosurgery
TGF-β	Transforming Growth Factor Beta
TNF-α	Tumor Necrosis Factor Alpha
TRT	Targeted Radionuclide Therapy
VMAT	Volumetric Modulated Arc Therapy
